# An automatic integrative method for learning interpretable communities of biological pathways

**DOI:** 10.1093/nargab/lqac044

**Published:** 2022-06-24

**Authors:** Nicasia Beebe-Wang, Ayse B Dincer, Su-In Lee

**Affiliations:** Paul G. Allen School of Computer Science and Engineering, University of Washington, Seattle, WA 98103, USA; Paul G. Allen School of Computer Science and Engineering, University of Washington, Seattle, WA 98103, USA; Paul G. Allen School of Computer Science and Engineering, University of Washington, Seattle, WA 98103, USA

## Abstract

Although knowledge of biological pathways is essential for interpreting results from computational biology studies, the growing number of pathway databases complicates efforts to efficiently perform pathway analysis due to high redundancies among pathways from different databases, and inconsistencies in how pathways are created and named. We introduce the PAthway Communities (PAC) framework, which reconciles pathways from different databases and reduces pathway redundancy by revealing informative groups with distinct biological functions. Uniquely applying the Louvain community detection algorithm to a network of 4847 pathways from KEGG, REACTOME and Gene Ontology databases, we identify 35 distinct and automatically annotated communities of pathways and show that they are consistent with expert-curated pathway categories. Further, we demonstrate that our pathway community network can be queried with new gene sets to provide biological context in terms of related pathways and communities. Our approach, combined with an interpretable web tool we provide, will help computational biologists more efficiently contextualize and interpret their biological findings.

## INTRODUCTION

Many computational biology studies aim to identify groups of genes that are associated with or differentially expressed with respect to phenotypes of interest (1,2). Performing pathway analyses to associate these gene-level findings with biological processes is a crucial step in providing both biological context for genes and a systems perspective to the analysis. Researchers have constructed *pathways* (i.e. networks of genes that interact in various ways to perform certain biological tasks (3)) using a variety of methods; these range from large-scale computational analysis over literature (e.g. Gene Ontology (4)) to hand-curation by experts (e.g. KEGG (5–7) and REACTOME (8)). These pathway gene sets are then commonly used in *pathway enrichment analysis*, where a new list of genes is compared with previously established pathway gene sets (via statistical tests to measure whether their genes overlap at higher than random rate) in order to identify which pathways are most related to the gene set of interest.

**Figure 1.**
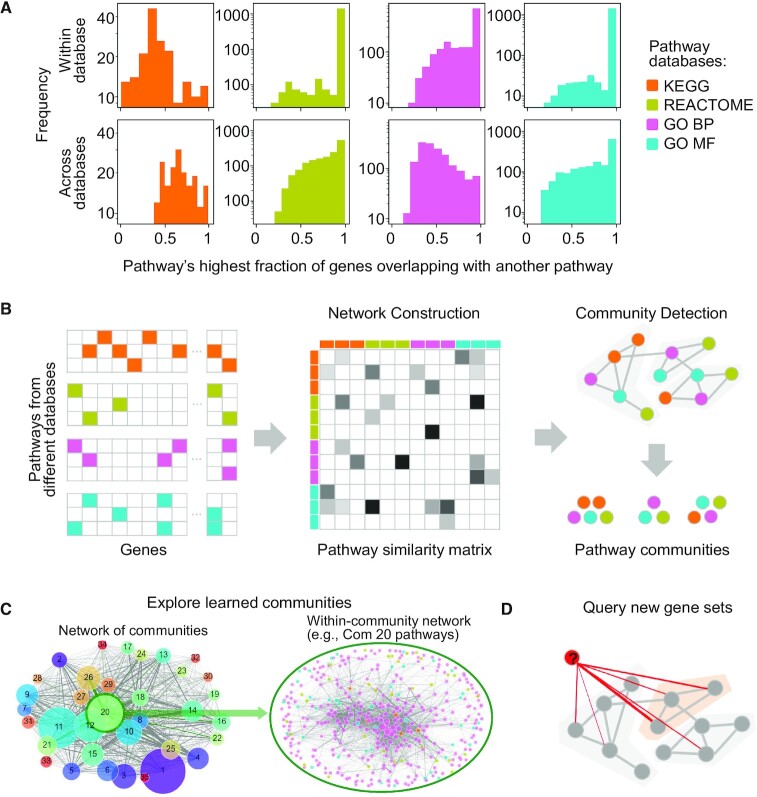
Overview of the pathway community approach. (**A**) The pathway overlap problem in pathway enrichment analyses. For each pathway database, we show the distribution of each pathway's maximum fraction of overlap with other pathways, both within the same database (top) and with other databases (bottom). (**B**) Our approach for learning pathway communities: we first construct a pathway network based on gene overlaps, and we then perform community detection to produce pathway communities. (**C**) Schematic highlighting of functionality on our webpage showing detailed views of each learned community. (**D**) Schematic highlighting of our proposed method for querying a new gene set against our learned communities to identify relevant processes enriched in a query gene set.

Since many pathway gene set databases offer unique advantages, it has become common practice to perform pathway enrichment analysis across multiple databases. Unfortunately, the difficulty of interpreting pathway-level results is increasing as the number of available pathways and databases grows. Figure 1A shows that pathways across different databases and even within a database often have highly overlapping gene sets, causing redundancy in enrichment results. In fact, 50% of all pathways in Figure 1A have some corresponding pathway in a separate database, with at least 70% of genes in common. Therefore, a single query of genes can return multiple enriched pathways (e.g. KEGG’s oxidative phosphorylation and Parkinson's disease pathways, which have 71% overlap), and it may be difficult to assess how the pathways are related, especially if they are from varying sources. Systematically analysing these phenomena can provide context for the simultaneous capture of multiple pathways.

Many studies have attempted to solve the problem of pathway redundancy. One common approach collapses pathways from multiple sources into a condensed set of high-level pathways – such as PathCards (9), MSigDB Hallmark (10) or GO slim (11) – to simplify enrichment tests; however, by condensing many pathways, these approaches tend to remove smaller nuanced pathways describing specific biological functions. Another set of approaches identify related pathways in a post-hoc manner with respect to specific queries. For example, Donato *et al.* (12) identify crosstalk effects when measuring enrichment of pathways for a set of differentially expressed genes, providing a *revised* set of enriched pathways with crosstalk effects removed. A similar approach has also been applied to identify broad patterns of co-occurring pathways in a collection of transcriptomic datasets (13). In these approaches, the relationships identified among pathways are with respect to collected data (e.g. a specific transcriptomic dataset), whereas a dataset-agnostic method relying only on the pathways themselves may provide a complementary approach for exploring related pathways in a unified and data-agnostic manner. To that end, pathway network visualization tools, such as Enrichment Map, have been developed to highlight the relatedness of pathways (14) but do not map pathways to functional categories. Although standard clustering algorithms have been useful for identifying groups of related pathways (15,16), they are limited in that these approaches have not been systematically validated with respect to expert labels. Furthermore, previous clustering-based approaches have relied on manual annotation of clusters and thus do not scale well to the growing numbers of pathways and databases (14–16).

**Figure 2.**
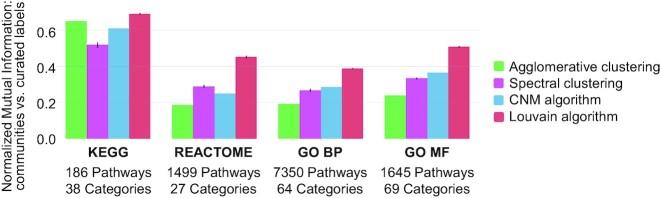
Comparison of graph clustering and community detection algorithms we evaluated. Pathway graphs and communities were learned separately for each database and then compared with curated category labels from each source database via normalized mutual information.

**Figure 3.**
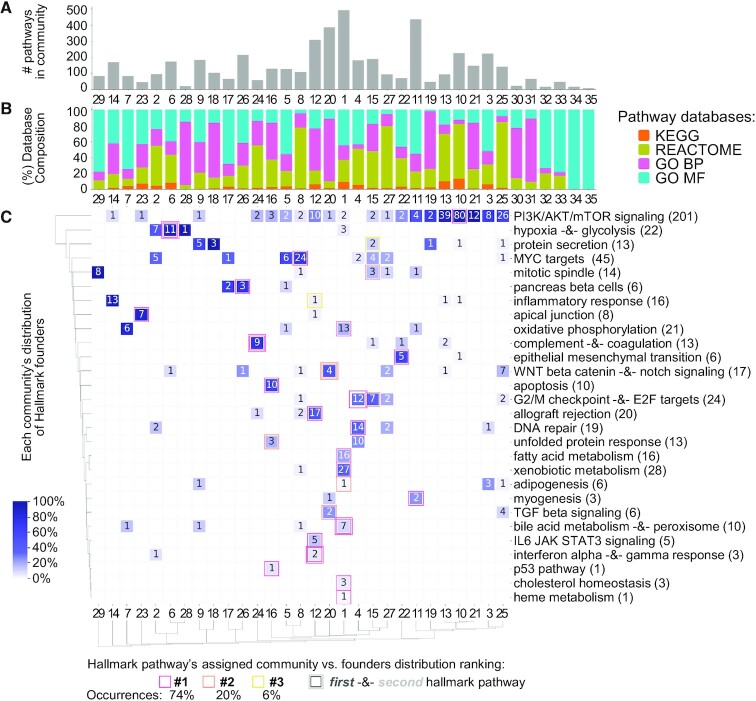
Overview of learned communities and their association with known processes. (**A**) Communities’ number of member pathways (ranging from 7 to 492 members). (**B**) Composition of pathway database members in each community. (**C**) Communities’ associations with MSigDB Hallmark pathways. Each Hallmark pathway has a list of associated ‘founder’ gene sets, some of which are in the REACTOME and KEGG databases. Heatmap annotations indicate the number of founders in each community associated with each Hallmark pathway. Cells are colored by each community’s frequency of founders distributed across Hallmark pathways (e.g. the darkest purple indicates that all Hallmark founders contained in a community are mapped to a single Hallmark pathway). We note that some Hallmark pathways have identical founder gene sets, and we include both Hallmark pathways in the same row of the heatmap (separated by ‘-&-’ in the label). Finally, we use PAC’s gene set querying method (described in Materials and Methods) to assign each Hallmark pathway to a community based on its member genes. The assigned communities are indicated by squares in the heatmap, and these squares are colored by how closely they agree with the top community based on founders.

**Figure 4.**
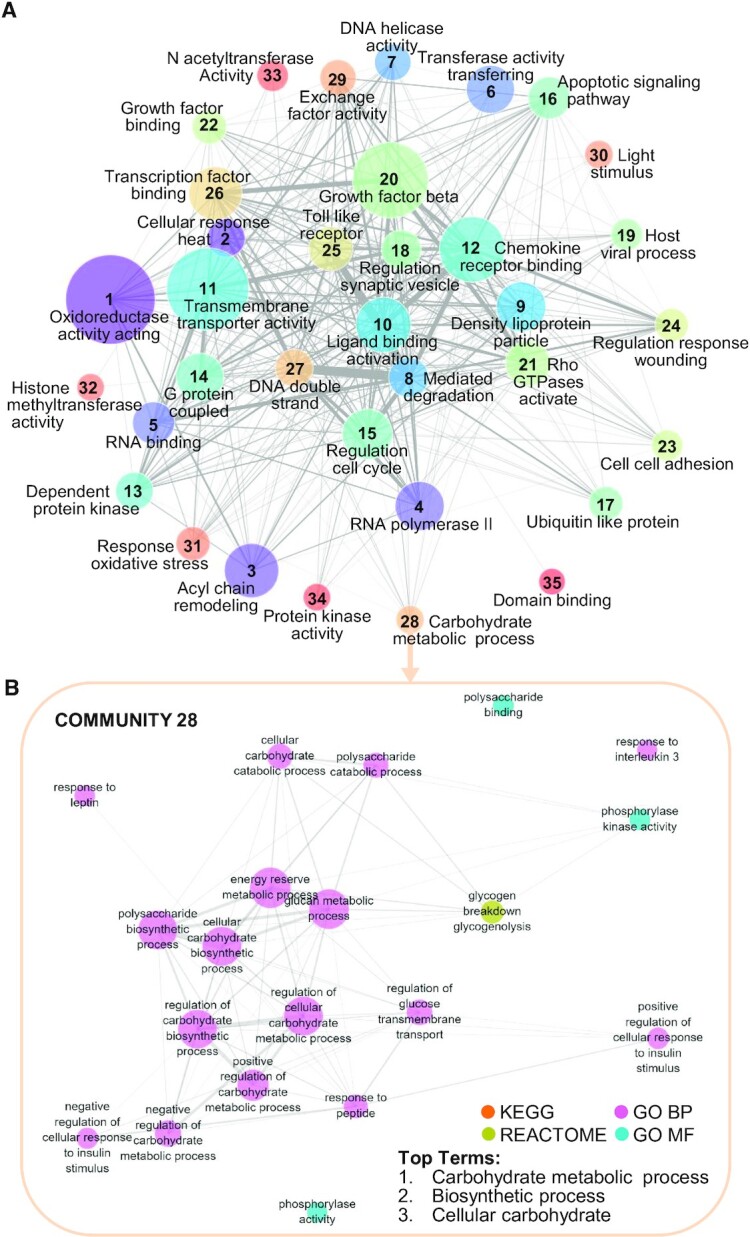
(**A**) The final learned community network. We display all community nodes, with sizes proportional to the number of pathway members, and edge widths proportional to the average weights among all pairs of edges between members of each community. Each community is labeled with automatically generated labels as described in Methods. (**B**) An example view of a single community. We display all members of community 28 as nodes with sizes proportional to their hubness in the subnetwork containing only community 28 pathways, and colored by the database for each pathway. Edge widths are proportional to the –log10(p-value) for Fisher's exact test measuring gene overlap between pathways.

**Figure 5.**
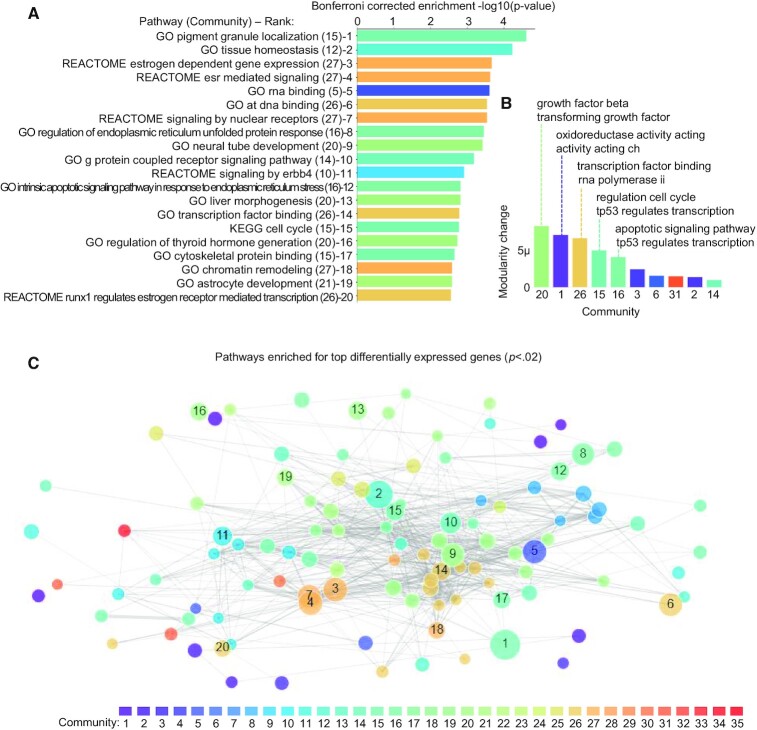
(**A**) Enrichment of top pathways for the 243 most significantly differentially expressed genes biopsied samples for ER + vs. ER- breast cancers. Each bar shows the –log10(p-value) for enrichment based on Fisher's exact test after Bonferroni correction over all pathways. Each pathway is labeled with its assigned community and enrichment rank. (**B**) Using the PAC’s gene set querying method (described in Methods), we query the 243 most significantly differentially expressed genes against the learned communities and display the modularity change associated with assigning the query gene set to each community. The top 10 communities are shown, with the top two automatically generated labels (Methods) for each of the top 5 communities, indicating processes that are generally related to the query gene set. (**C**) Network overview for the top 108 enriched pathways (*P*< 0.02) for the 243 genes described above. Each node is colored by its community and sized proportionally to the enrichment –log10(p-value), and we annotate the top 20 pathways with their rank as indicated in (A).

In this paper, we address the problems of heterogeneity and redundancy across literature-derived pathways by introducing the Pathway Communities (PAC) framework. Our framework uniquely relies on the Louvain community detection algorithm to cluster pathways into communities based on their gene-level similarities (Figures 1 and 2). Further, we enhance the biological interpretability of cross-database pathway analysis by (i) characterizing learned communities with respect to pre-defined categories (Figure 3), (ii) devising a method to algorithmically annotate communities (Figure 4A), (iii) applying interactive visualization techniques to investigate newly revealed connections within and across pathway communities (Figure 4) and (iv) providing a tool to help researchers investigate novel gene sets in the context of our learned communities and member pathways, which we demonstrate on a breast cancer gene expression example (Figure 5).

## MATERIALS AND METHODS

### Pre-processing of pathways and generation of curated categories

For our analyses, we constructed a pathway graph comprised of pathways from KEGG (7), REACTOME (8) and Gene Ontology (GO) (4) gene sets, downloaded from MSigDB v7.0 (17,18). Each pathway database contains a list of named pathways that each have set of associated genes. For each database, we additionally pre-processed a provided set of curated categories or hierarchical relationships among pathways to identify higher-level categories associated with each pathway (see Supplementary Methods). These simple mappings from pathways to curated categories were treated as ground-truth labels for evaluating our method. Although these curated categories share some common themes across databases, it is not possible to combine them, so initial validation experiments were performed separately for each database. Finally, we downloaded MSigDB Hallmark gene sets (10), a collection of refined pathways meant to summarize thousands of founder gene sets (some of which are KEGG and REACTOME pathways in our analysis), which we use for additional validation.

### Generating the pathway graph and learning communities

Using the PAC method for identifying communities of related pathways involves two steps: 1) construction of the pathway network, and 2) detection of pathway communities (Figure 1B). For the first step, we represented pathways from multiple databases as a large graph, where each node is a pathway (with an associated set of genes). For each pair of pathways, we performed Fisher's exact test which evaluates the significance of their gene overlap under a hypergeometric null distribution (Supplementary Methods andSupplementary Figure S1A) (19–21). We added edges between all pairs of pathways, with edge weights corresponding to the –log_10_(*P*-value) from Fisher's exact test measuring the significance of gene overlap (*P*-values were Bonferroni corrected, and edges between pathways with *P* > 0.01 were set to a weight of 0). This process generated a sparsely connected graph in which each node represents a pathway, and edges indicate similarity of pathways with respect to shared genes.

For the second step, we identified communities of related pathways from this network using the Louvain community detection algorithm (22,23) using the Community API in Python. To our knowledge, ours is the first work to use an approach based on graph modularity to cluster pathways. The Louvain algorithm learns well-connected communities from a network by greedily optimizing for graph modularity, namely, a measure of the density of intra- vs inter-community edges. During our evaluation, we first performed the PAC method separately for each pathway database (generating four separate pathway graphs and performing community detection separately). We ran the Louvain algorithm with a resolution parameter of 0.4 and conducted several experiments to verify the stability of our approach with different initializations, hyperparameters, and alternative methods for graph construction and evaluation (Supplementary Methods; Supplementary Figures S2–S6). We also compared other graph clustering approaches with the Louvain algorithm: agglomerative clustering, spectral clustering, and the Clauset-Newman-Moore (CNM) algorithm (another modularity-based approach); for all methods, we evaluated several hyperparameter options. Figure 2 reports the highest NMI achieved between the learned clusters and curated categories (i.e. the reduction uncertainty for the curated category label when the algorithmically learned community label is known).

Finally, after confirming the consistency of our learned communities with curated categories within each database (Figure 2), we learned an integrative set of communities from the pathway graph that combined all four pathway databases, resulting in 35 learned communities across 4847 pathways. The full list of pathways’ community assignments is provided in Supplementary Table S1. Our code for producing the pathway graph and learning communities is available at https://gitlab.cs.washington.edu/nbbwang/P-COM.

### Automatic labelling of pathways

To automatically generate descriptions for our communities that were independent of expert-curated category labels, we developed a method based directly on the names of member pathways. For each community, we pre-processed member pathway names from MSigDB and identified commonly appearing terms. After pre-processing these names to remove database identifiers and common stop words (using the Natural Language Toolkit Python package), we counted instances of all 3-mers across community members and used the most commonly appearing 3-mer (with at least three appearances) as a label to describe our community. In the case of a tie, we selected the 3-mer whose source pathways had the highest average hubness within their community. When no 3-mers made at least three appearances, we repeated the process with 2-mers. Supplementary Figure S7 shows an example of this procedure, and Supplementary Table S2 provides the top ten terms for each community.

### Querying new gene sets

As described above, the PAC method creates a graph with edges based on gene set overlaps among pathways and learns communities via the Louvain algorithm, which greedily optimizes the modularity of graph partitions. Therefore, using the following process, it is straightforward to query a new gene set in our learned community network. First, we calculate Fisher's exact test *p*-values between the new gene set and each pathway already in the graph (Supplementary Figure S1B), and we then temporarily add the gene set as a new node in the pathway graph. Next, holding the previously learned partition constant and initially considering the new gene set as a single-member community, we calculate the modularity change associated with moving the gene set to each of the other communities. We then rank the candidate communities for the new query gene based on their associated increase in modularity.

To ensure that this approach is useful for any arbitrary list of genes (e.g. from a differential gene expression analysis), we first validated it by again using the MSigDB Hallmark pathways, because some of their founders are pathways in our graph. Thus, we evaluated whether, when queried, these Hallmark gene lists tended to be assigned to communities containing many of their founder gene sets. Finally, as described below, we created an interactive web tool to help users query a list of genes and identify candidate communities most closely associated with it.

### Interactive web tool

Because the pathway graph contains thousands of pathways and learned communities contain up to 492 pathways, our integrative communities are not amenable to static visualizations. Therefore, we created an interactive web tool, available at https://pathwaycommunities.cs.washington.edu/, using the Plotly Dash framework. The tool lets users explore the community and pathway networks to gain a clearer understanding of how pathways relate to each other and the higher level processes learned by each community. It offers several views. First, a *community-level view* shows users the community graph (i.e. Figure 4A) along with detailed annotations, including the top automatically generated labels, pathway members, and most commonly appearing genes. Users can query specific biological processes (i.e. automatically learned labels) to identify which communities contain relevant pathways. Second, a *pathway-level view* reveals sub-graphs containing all pathway members for a selected community. Here, users can highlight a specific pathway in the graph to visualize how it relates to other pathways in its community. Third, a *gene-level view* helps users query any gene to see which communities contain pathways with that gene and whether the gene appears disproportionately often in certain communities (Supplementary Table S3 provides the full list of genes with significant overrepresentation in any community as described in Supplementary Methods). Finally, we provide a page that lets users *query a new gene set* against our pathway network and visualize both enriched pathways and top related communities (based on the method described above). Together, these different pages for examining the learned communities of pathways, along with the ability to query a new gene set, may allow users to more effectively interpret their biological findings in the context of known pathways.

### Biological example with breast cancer data

To demonstrate an application of our method to new gene sets, we used an example of a differential gene expression analysis in breast cancer samples. Data was provided by the Molecular Taxonomy of Breast Cancer International Consortium (METABRIC) database. The database includes breast cancer sequencing data from 2,509 patients; gene expression measurements from biopsied breast cancer samples, including 24 368 measured genes; and phenotypic or treatment labels associated with each sample (e.g. estrogen receptor status and whether the patient was treated with chemotherapy).

We restricted our analysis to 2469 sampled profiles for which estrogen receptor status was reported, of which 74% of samples were positive for estrogen receptors. For each of the 24 368 genes measured, we compared expression levels for ER + and ER- samples using two-sided independent *t*-tests and identified 8,984 genes significantly differently expressed between groups (*P* < .05 after Bonferroni correction; Supplementary Methods describe data processing). Consistent with prior knowledge (24), this indicates that expression differences between ER + and ER− cancers are widespread across genes. To refine our understanding of the top genes, we identified the top 1% (243) of genes with the most significant differential expression (Supplementary Figure S8) and examined them in the context of our community network, thus enhancing the interpretability of a set of differentially expressed genes.

## RESULTS

### The PAC framework uses a Louvain community detection method that outperforms alternative approaches

To evaluate whether we could learn informative and meaningful communities from pathway networks, we separately applied the pathway network construction step to each pathway database and then applied various community detection and clustering algorithms to each individual graph. We then computed the normalized mutual information (NMI) to compare the resulting communities with the curated categories (see Methods for details) from each database. Figure 2 shows that the Louvain community detection method achieved high NMI scores when comparing the automatically learned communities with ground-truth curated labels, exceeding NMI scores from all alternative approaches across all pathway databases (*P* < 0.001 for all *t*-tests; alternative evaluation metrics revealed similar results, as shown in Supplementary Figure S2). This indicates that the Louvain community detection method generates communities that are consistent with expert-curated labels and offers a promising approach for automatically categorizing communities based on their shared genes.

### PAC combines four major pathway databases, resulting in communities that are consistent with hand-curated categories

To learn a unified set of communities across all 4847 gene sets from the four different sources (KEGG, REACTOME, GO BP and GO MF), we constructed a joint network and used the Louvain algorithm with a resolution of 0.4 (selected because it provided high NMI scores across all four datasets; Supplementary Methods & Supplementary Figure S4). This approach generated 35 communities, ranging in size from 7 to 492 pathways (Figure 3A) and effectively integrated pathways from different sources (Figure 3B), which indicates that the communities are likely driven by function rather than database-specific signals.

Like the separately generated ones, communities found from our combined pathway graph tend to be very consistent with their own curated categories (NMIs of 0.62, 0.43, 0.32, and 0.41 for KEGG, REACTOME, GO BP and GO MF, respectively; NMI of 0.30 when all curated categories were concatenated; Supplementary Figures S9-1S3 show comparisons between these communities with curated categories). However, differing curated categories from each database are not easily reconcilable into common biological themes. Thus, to explore whether the learned communities capture meaningful common processes, we analysed our results with respect to the MSigDB’s Hallmark pathways (v7.1), developed to summarize pathways across MSigDB’s various sources (10).

All Hallmark pathways have associated sets of *founder pathways*, sets of pathways from which the Hallmark pathways are derived, including 745 pathways in our combined graph. We initially observe that our automatically learned communities are highly consistent with grouping pathways by Hallmark pathways for which they are founders (NMI = 0.60; pathways that were not Hallmark founders were not considered in this calculation). Furthermore, purple cells in Figure 3C illustrates the distribution of Hallmark pathway founders within each community; most of our communities are associated with few Hallmark pathways, signifying that Hallmark pathways provide insight into coherent biological processes within communities. For example, there is a nearly one-to-one mapping between community 23 and the Hallmark ‘apical junction’ pathway (i.e. genes involved in adherens and tight junctions between cells (10)), suggesting that this Hallmark pathway may be an appropriate annotation for community 23. This is further supported by the fact that most pathways in the community relate to cell-to-cell adhesion (Supplementary Table S1), consistent with the biological function of the Hallmark pathway. Similarly, all Hallmark apoptosis founders are in community 16, consistent with the fact that many central members of the community relate to apoptotic signalling (Supplementary Table S1).

Although the examples illustrated above and in Figure 3C are promising, because Hallmark pathways are based on only a small subset (15.4%) of our original pathways, they cannot be used to annotate smaller communities with no Hallmark founders. Furthermore, this interpretation may fail to reveal highly specific processes occurring within the communities. For example, the PI3K/AKT/mTOR Signalling Hallmark Pathway, broadly important in regulating the cell cycle, is split across several of our communities, showing that these communities may each represent more specific sub-processes. In the next section, we address this problem by automatically generating descriptive labels for each community.

Finally, we also use Hallmark pathways to validate our method for querying new gene sets in the PAC framework. Because each Hallmark pathway has a set of associated founder gene sets (some of which are in our pathway network), we use our querying method to assign each hallmark pathway to a community in our network; we can then determine if there is agreement between the community assigned via our method and communities containing its founders. For 74% of Hallmark pathways, Figure 3C shows that the assigned community based on our querying method is the same community that contains the most founders (see pink cell outlines in Figure 3C); for all Hallmark pathways, the assigned community is in at least the top three communities based on founder membership.

### PAC’s Automatically generated labels and visualizations provide high-level overviews of learned communities

To highlight high-level relationships among our pathway communities, we visualize our pathway community graph with their automatically learned labels (Figure 4A). First, we observe that many of our automatically generated label terms are consistent with our Hallmark pathway analyses (Figure 3C), e.g. Community 16, whose top label was ‘Apoptotic signalling pathway,’ is consistent with its most closely related Hallmark pathway, ‘apoptosis.’ Importantly, our labelling approach also provides insights unavailable from Hallmark pathway analysis alone. For example, Communities 9, 18, and 19 are all strongly associated with the Protein Secretion Hallmark pathway (Figure 3), but our automatically generated labels reveal separate phenomena (i.e. lipoproteins, synaptic processes, and viral activity for Communities 9, 18 and 19, respectively). Overall, Figure 4A demonstrates that our communities’ labels cover a broad range of cellular activities while remaining sufficiently specific to describe well-defined biological functions, while network edges highlight the interrelatedness of biological processes across these communities.

Finally, to demonstrate that our approach produces informative pathway communities, we explored individual networks within communities. As an example, Figure 4B visualizes Community 28, one of multiple communities associated with the Hallmark hypoxia and glycolysis pathways. Pathway members are consistent with the top label ‘carbohydrate metabolic process,’ and the community integrates pathways related to this biological function from multiple databases. These promising results highlight that our process can overcome database bias and capture functional similarities.

Supplementary Table S1 shows community pathway membership, and Supplementary Table S2 shows the top labels for each community. Our web tool provides detailed interactive visualizations for each community; see https://pathwaycommunities.cs.washington.edu/.

### A biological example: genes differentially expressed between estrogen receptor positive versus negative breast cancers relate to relevant pathway communities

Breast cancers are commonly classified by their estrogen receptor (ER) status—i.e. whether estrogen receptors are expressed in cancer cells (ER+) or not (ER−). These cancer subtypes manifest in markedly different ways and require different treatment regimens because ER+ cancers rely heavily on estrogen to grow and reproduce, whereas ER- cancers do not (25,26). Thus, ER status is associated with a wide range of transcriptional differences between ER + and ER− cancers (27). In this example, we conduct a simple differential expression analysis to identify the most differentially expressed genes and then highlight how our method clarifies these findings. In particular, as described in Methods, we identify the top 1% of the most significantly differentially expressed genes between 1,825 ER + and 655 ER- samples from the METABRIC dataset (Supplementary Figure S8) and use these genes as a query gene set to explore relevant pathways in the context of our learned communities.

Figure 5A shows the top enriched pathways (as determined by Fisher's exact test of overlap for the top genes and all pathways), which would be the final outcome of a standard pathway analysis. By additionally colouring each bar with the community to which the pathway was assigned, we see that some communities appear frequently in the top 20 enriched pathways.

Next, we query the top genes against our communities (see Materials and Methods). This approach considers not only the top enriched pathways, but the overall association of all pathways in each community with the query gene set. This analysis reveals that the top communities related to differentially expressed genes between ER+ and ER− broadly relate to growth factors and cell differentiation (community 20) and metabolic activity (community 1) (Figure 5B). Interestingly, although community 27 contains multiple estrogen-related pathways that are highly enriched in the top genes (e.g. #3 and 4 in Figure 5A), most pathways in community 27 relate to broader cell-cycle activity (Supplementary Tables S1, S2); therefore, that community is not favoured in the community-level analysis. Thus, although our approach identifies specific pathways relevant to our gene sets, use of the community network query reveals some broader patterns of processes captured in the top genes related to ER-status. Finally, Figure 5C shows a network visualization that highlights 108 pathways enriched at the *P* < 0.02 level in our gene set query. This helps the user visualize not only which pathways are enriched but also how they relate to each other (note that interactive versions of Figure 5B, C, which provide more detail, are available at https://pathwaycommunities.cs.washington.edu/).

## DISCUSSION

Our approach is not without limitations. In particular, because our graph is constructed using Fisher's exact test-based edges, our method for querying new gene sets also relies on the use of Fisher's exact test for pathway enrichment to be consistent with the rest of the graph's structure (since querying a new gene set involves simulating its addition as a node to the pathway network). Thus, our tool does not currently support the use of an alternative approach to compute pathway enrichment (e.g. GSEA); we may implement this functionality in the future. Additionally, although we empirically found that our automatic community annotation approach was consistent and interpretable, it relies primarily on the quality of pathway names. If the analysis were repeated with a new set of pathway databases that used uninformative pathway names, our approach for automatically labelling communities would not be applicable.

In summary, we contribute the PAC framework, an automated approach for identifying communities of closely related pathways across several databases that successfully recapitulates expert-curated categories. By conducting separate analyses on pathway databases, we verified that the learned communities were consistent with curated ground truth labels. When scaled up to an integrative analysis across four databases, we verified that learned communities were consistent with Hallmark pathways. Unlike previous methods focused solely on visualization of pathway enrichment results, we leverage an automatic labelling approach to yield additional insights about biological pathway relationships. We also found that maintaining a pathway-level understanding of our communities provides additional nuance and context that is lost by consolidated gene sets (e.g. using Hallmark pathways alone for pathway enrichment analysis). Further, our approach may also complement existing methods of post-hoc disambiguation (e.g. by Donato *et al.* (12)), as our learned communities highlight closely-related pathways which would likely be combined and revised with respect to a specific dataset. Finally, we believe that our tool to query new gene sets against our analyses (demonstrated with a breast cancer gene expression data example analysis) and our interactive webpage for examining relationships among pathways and communities will help computational biologists contextualize meaningful new findings for a wide variety of biological processes.

## DATA AVAILABILITY

Molecular Signatures Database (MSigDB) is a collection of annotated gene sets, which include KEGG, REACTOME, GO, and Hallmark gene sets (http://www.gsea-msigdb.org/gsea/downloads.jsp#msigdb). Curated categories for KEGG pathways are available at https://www.genome.jp/kegg/pathway.html. Curated categories for REACTOME pathways are available from their interactive web browser (https://reactome.org/PathwayBrowser/). GO relations are provided at http://geneontology.org/docs/download-ontology/ (we used the go-basic version).

Data from the METABRIC (Molecular Taxonomy of Breast Cancer International Consortium) cohort are available from the cBioPortal for cancer genomics. The specific dataset used in our study was downloaded here: https://cbioportal-datahub.s3.amazonaws.com/brca_metabric.tar.gz, and an interactive view of clinical features is available here: https://www.cbioportal.org/study/summary?id=brca_metabric.

Python packages used in this study include the Community API (python-louvain.readthedocs.io/), NetworkX (https://networkx.org/), the Natural Language Tool Kit (https://www.nltk.org/) and Scikit-learn (https://scikit-learn.org/stable/).

Our code for reproducing our results is available at https://gitlab.cs.washington.edu/nbbwang/P-COM. Our interactive webpage accompanying this paper is available at https://pathwaycommunities.cs.washington.edu/.

## Supplementary Material

lqac044_Supplemental_Files
