# Supplementary material for: An automatic integrative method for learning interpretable communities of biological pathways
Source: NAR Genom Bioinform. 2022 Jun 24;4(2):lqac044. doi: 10.1093/nargab/lqac044 (PMC9228877; doi:10.1093/nargab/lqac044)

SUPPLEMENTARY METHODS

Pathway graph construction

In the PAC method, we create a pathway network in which each node represents a pathway, and each edge represents how closely two pathways are related in terms of their shared genes. In order to represent relatedness of pathways, we use Fisher's exact test (FET), which is a commonly used statistical test for measuring gene set enrichment and relatedness between pathways[1]. To that end, we first generated a reference set of 17,640 genes (G) which were present in at least one pathway in any of the four pathway databases. For each pathway i we have P_i, the set of all genes in the pathway, and P_i^c, the set of all genes not in the pathway (i.e., in the reference set G but not P_i). For a pair of pathways Pi and P_j, we therefore use Fisher's exact test based on the contingency table of these two pathway gene sets, as shown in Supplementary Figure 1A. Finally, we use the –log10(p-values) from each pairwise Fishers exact test (after Bonferroni correction across all pairs of pathways) as edges in the graph (note that edge weights are set to 0 for pairs of pathways that were not significantly overlapping at the p<.01 level). As described by Rivals et al. (21), several formulations of enrichment tests all rely on an underlying hypergeometric null distribution, and all of these may equally be called Fisher's exact tests or hypergeometric tests. For our purposes, we used the python scikit-learn implementation of FET. In particular, we use two-sided tests to be consistent with the fact that they are most commonly appropriate in practice for evaluating the enrichment or depletion of pathways in differential gene expression analyses (e.g., our example using breast cancer data) (21).

Preprocessing of pathways' curated hierarchies

While KEGG assigns each of its pathways to a high-level category that we use directly, REACTOME, GO Biological Processes (BP) and GO Molecular Function (MF) provide a tree hierarchy for their pathways that we trace to define the associated highest-level categories (see Figure 2 for the number of pathways and categories from each database). The processing steps below yield a set of "curated" category labels for pathways in each of the four databases, listed in Supplementary Table 1, which we use to evaluate our community detection methods:

KEGG: KEGG consists of 186 pathways, which are divided across 35 higher-level categories provided by the resource, and thus needed no further pre-processing

REACTOME: REACTOME consists of 1,499 pathways which are related to each other as a hierarchy of pathways ranging from highly specific to 25 general pathways (as shown on their interactive webtool: https://reactome.org/PathwayBrowser/). For each pathway, we trace the hierarchy tree and assign the pathway's category as the highest-level pathway to which we can trace the original pathway. A small number of pathways have multiple parents, and in these cases, we count all possible paths to higher-level categories, and select the category label for which there are the most paths.

Gene Ontology (GO): GO consists of three subcategories, which we treat as separate sources: Biological Process (BP), Cellular Component (CC), and Molecular Function (MF) containing 7,350, 1,001, and 1,645 gene sets, respectively. For this analysis, we exclude the CC gene sets because unlike the other databases and GO categories which relate more closely to biological processes, GO CC relates more closely to cellular structures. The GO resource contains detailed mappings (of several types) among gene sets which form a hierarchy, and only considered the 'is_a' relations between gene sets which were the most frequent relation type and we discarded the 'obsolete' GO terms that are not connected to any other terms. For BP and MF gene sets separately, we traced the entire hierarchy of gene sets mapping each gene set to its direct parents. Since GO terms often have multiple parents, we traversed the hierarchy multiple times for all possible traversals from a leaf node to the high-level node and recorded the most frequent curated category for each level of the tree for each gene set. This pipeline returned us a multiple layer of hierarchy and to define a universal set of curated labels, we selected the highest possible level in these hierarchies with a reasonable coverage, obtaining 64 and 69 high level categories for GO BP and MF, respectively.

Community detection: Stability and Robustness

The Louvain algorithm employs a greedy approach, and outcomes are dependent on an initial ordering of nodes. Thus, we repeatedly ran the algorithm with different initializations to explore the stability of the resulting communities in the full graph. Using a resolution of 0.4 as described above, we find slight variations in learned communities, however find overwhelmingly similar results, as shown in Supplementary Figure 5. When running the algorithm 100 times, we found that the median pair-wise NMI of learned communities between runs was 0.84, and all pairs had NMIs above 0.77.

We further examined alternative approaches for computing edges in our community graph, including using the Jaccard similarity coefficient, and overlap coefficient. These approaches tended to produce similar or slightly worse downstream community detection results compared with FET-based edges; however, the Louvain algorithm continued to be the best community detection approach even with alternative edge construction methods (Supplementary Figure 3).

Although the resolution of 0.4 was selected because it tended to yield the highest agreement with curated categories (Supplementary Figure 3), and produced a relatively easy-to-interpret 35 communities on the full pathway set, we also explored alternative resolutions for our final community detection analysis and found that the communities tended to agree with each other (although lower resolution-based communities tended to be subsets of larger communities learned in for higher resolutions; Supplementary Figure 6).

Identifying significantly overrepresented genes in communities

For each community, we identified genes which we believe are disproportionately represented across member communities. For each gene-community pair, we use one-way chi square tests to calculate whether the number of pathways in the community that contain the gene appears at a different rate than if the gene were randomly distributed across all pathways. We then consider genes to be significantly

overrepresented genes to be those with a positive chi-square statistic and p<.01 after Bonferroni correction over communities and genes. Users may query genes to see whether they are significantly overrepresented in any communities on the *gene-level* view of our webpage at https://nicasia.github.io/PAC, and all results are available in Supplementary Table 3.

Data processing supplementary info for breast cancer data

We used data provided by the Molecular Taxonomy of Breast Cancer International Consortium (METABRIC) database, which we downloaded from the cBioPortal. In total, the dataset consists of gene expression profiles for primary tumors from 2,509 patients. Gene expression levels are reported for 24,368 genes, and phenotypic or treatment labels were also provided with each sample. To demonstrate the use of our tool with minimal overhead, we used the gene expression data directly as it was provided cBioPortal with no further pre-processing.

For our analyses, we restrict our analysis to 2,469 sampled profiles for which estrogen receptor status was reported, of which 74% of samples were positive for estrogen receptors. For each of 24,368 genes (labeled by HUGO gene symbols) measured, we compare expression levels for the 1,825 ER+ and 655 ER- samples using two-sided independent *t*-tests. After Bonferroni correction accounting for testing all 24,368 genes, we found that 8,984 genes were significantly differently expressed between groups. Of these genes, we use the 243 (top 1%) of genes with the lowest significant p-values (Supplementary Figure 8) for our enrichment analyses.

Supplementary Figure 1. Contingency tables used for Fisher's exact tests. (A) Contingency tables used to calculate pairwise pathway similarity among all pathways. Each pathway P_i is a set of genes, and we consider P_i^C to be the set difference between G (our reference gene set) and P_i. (B) Contingency tables used to query new gene sets (such as differentially expressed genes in our Breast Cancer example).

A

		Pathway *j* members		
		Yes	No	Total:
Pathway *i* members	Yes	$\lvert P_i \cap P_j \rvert$	$\lvert P_i \cap P_j^C \rvert$	$\lvert P_i \rvert$
	No	$\lvert P_j \cap P_i^C \rvert$	$\lvert G - (P_i \vee P_j) \rvert$	$\lvert P_i^C \rvert = \lvert G \rvert - \lvert P_i \rvert$
	Total:	$\lvert P_j \rvert$	$\lvert P_j^C \rvert = \lvert G \rvert - \lvert P_j \rvert$	$\lvert G \rvert$

B

		Differentially expressed genes		
		Yes	No	Total:
Pathway *i* members	Yes	$\lvert P_i \cap DE \rvert$	$\lvert P_i \cap DE^C \rvert$	$\lvert P_i \rvert$
	No	$\lvert DE \cap P_i^C \rvert$	$\lvert G - (P_i \vee DE) \rvert$	$\lvert P_i^C \rvert = \lvert G \rvert - \lvert P_i \rvert$
	Total:	$\lvert DE \rvert$	$DE^C = \lvert G \rvert - \lvert DE \rvert$	$\lvert G \rvert$

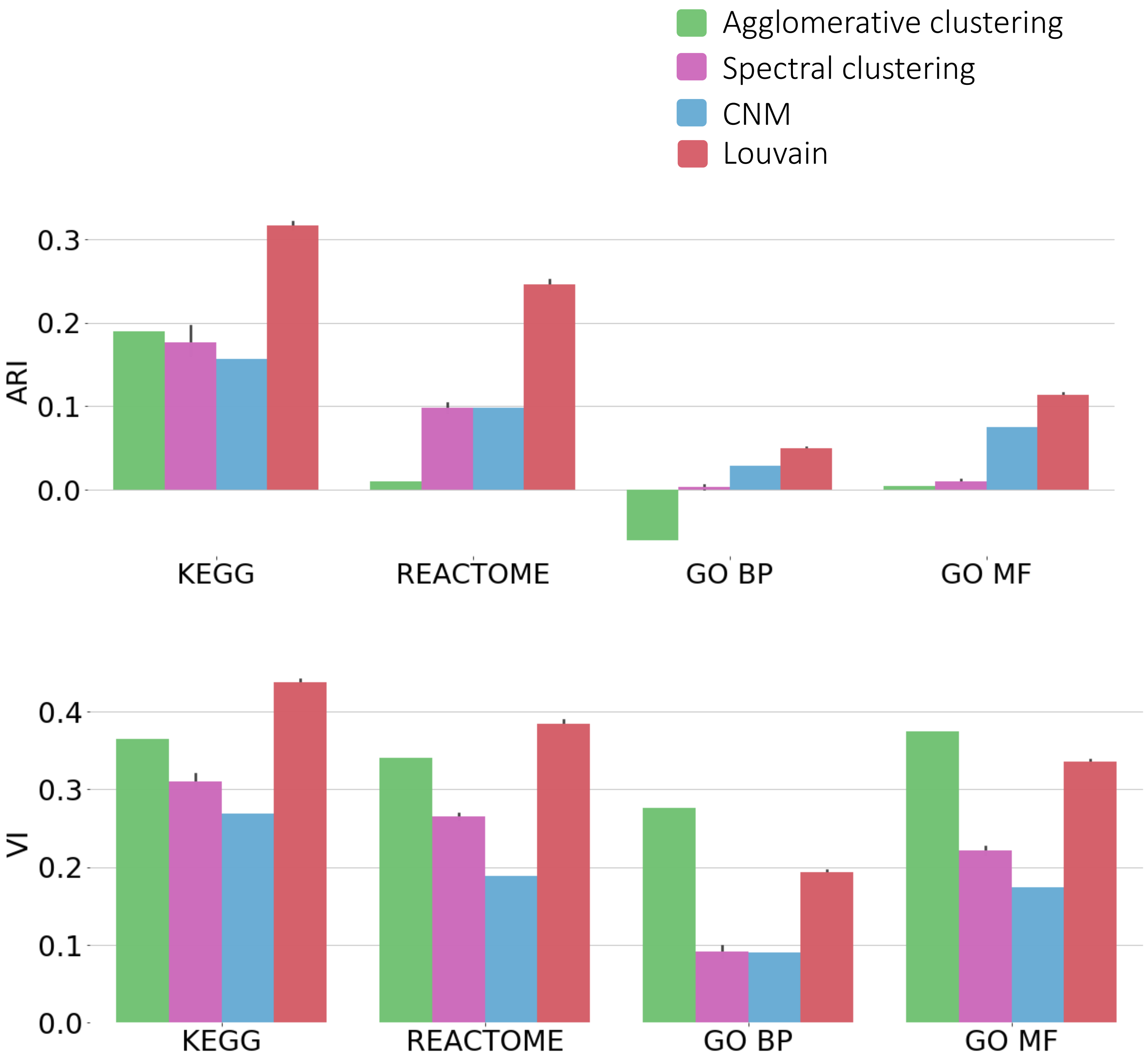

Supplementary Figure 2. While our main analyses use normalized mutual information (NMI) to compare methods, other metrics are available for evaluating clusters. We show two alternative evaluation metrics: adjusted Rand index (ARI) and variation of information (VI).

Supplementary Figure 3. (A) Comparison of downstream community detection performance for three different edge types in the pathway network. For each edge type, the pathway network is constructed based on the edge type described in the title of the table separately for each pathway database. We next perform the same four community detection methods on each of the four pathway databases and compare assignments to our ground truth labels for each community. (B) Comparison of Louvain community detection for alternative edge types using all pathways. While our method uses network edges calculated from Fisher's exact test, we also evaluate the similarity of our results compared with Jaccard index-based edges and overlap-coefficient base edges. For each edge calculation method, we run 100 rounds of the Louvain algorithm and compare pairwise normalized mutual information between all pairs of runs from FET-based edges to the alternative approach.

A.

NMI results using Fisher's Exact Test Edges

Methods	Kegg	Reactome	GO BP	GO MF
Agglomerative	0.65	0.1866	0.1923	0.2396
Spectral	0.6330	0.3265	0.2984	0.3239
CNM	0.6099	0.2496	0.2865	0.3661
Louvain	**0.6911**	**0.4484**	**0.3890**	**0.5106**

NMI results using Jaccard Index Edges

Methods	Kegg	Reactome	GO BP	GO MF
Agglomerative	0.6544	0.1875	0.1924	0.2398
Spectral	0.6334	0.2464	0.2985	0.3344
CNM	0.4008	0.0668	0.1249	0.1220
Louvain	**0.6841**	**0.3495**	**0.3714**	**0.4398**

NMI results using Overlap Coefficient Edges

Methods	Kegg	Reactome	GO BP	GO MF
Agglomerative	0.661	0.1837	0.2011	0.2310
Spectral	0.6420	0.2785	0.3132	0.1186
CNM	0.4164	0.0880	0.1208	0.35
Louvain	**0.6705**	**0.4573**	**0.3711**	**0.5050**

B.

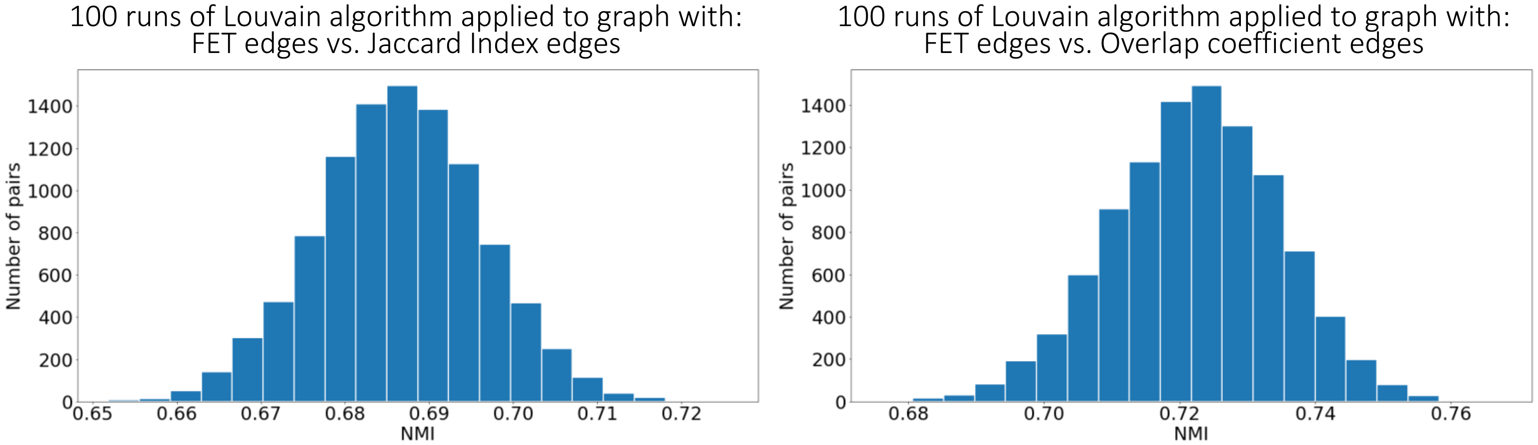

Adjusted rand index

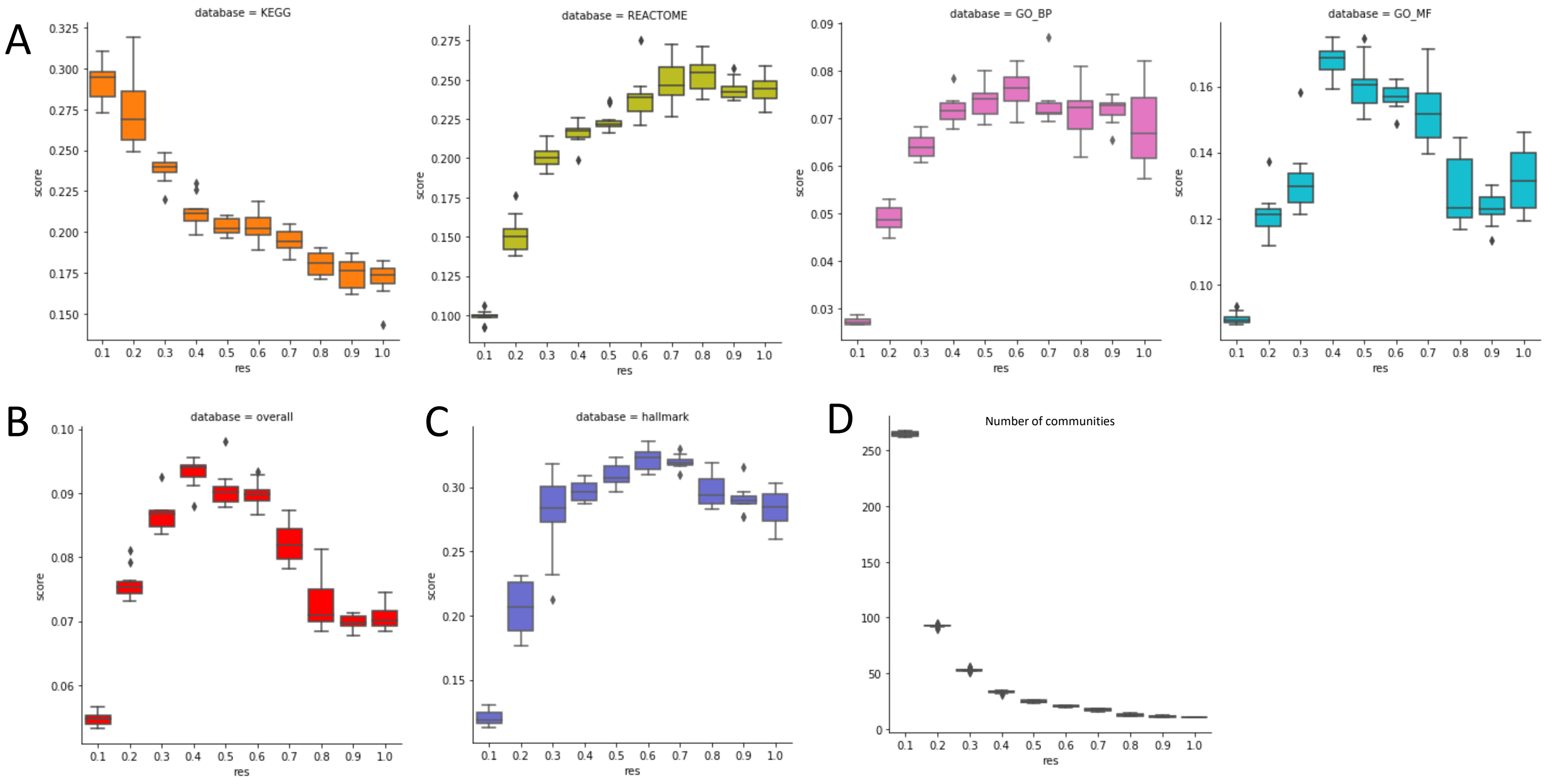

Supplementary Figure 4. Comparison of full graph Louvain community detection at different resolutions with curated categories. For the graph of 4,847 pathways from 4 pathway databases, we perform ten random runs of the Louvain community detection algorithm at resolutions ranging from 0.1 to 1, and for each run, obtain a set of learned communities. We then compare the assigned communities with curated categories. (A) Separately for each pathway database, we evaluate the adjusted rand index with curated category labels (ignoring all pathways outside of the database). We note that ARI was used because it is sensitive to cluster sizes. (B) We combine all 198 curated category labels from each of the four databases and compare our learned communities to these combined labels. (C) Among the 550 pathways that are founders of any Hallmark pathway, we compare the learned community label with labeling by Hallmark pathways to which each pathway was a founder. (D) Sizes of each community. Each subplot shows box-and-whisker plots based on the 10 repeated trainings for each resolution.

Supplementary Figure 5. Consistency of Louvain communities over random initializations. We ran the Louvain algorithm 100 times on the full pathway network using a resolution of 0.4. (a) From the 100 sets of Louvain community assignments, the distribution of all pairwise normalized mutual information (NMI) scores between all pairs of runs. (b) Frequency of the most frequent k-mer for each pathway across 100 runs. Blue lines indicate the first and third quartile; red line indicates the median frequency of most frequent k-mers across all pathways. (c) A more relaxed version of part b. For each pathway, we identify the top three k-mer labels from each run, and then aggregate these to identify the top three k-mers which appeared most commonly in top-three labels across all runs. We then compute, for each pathway, how many runs had any top three k-mer labels from that run overlapping with the top three overall k-mer labels.

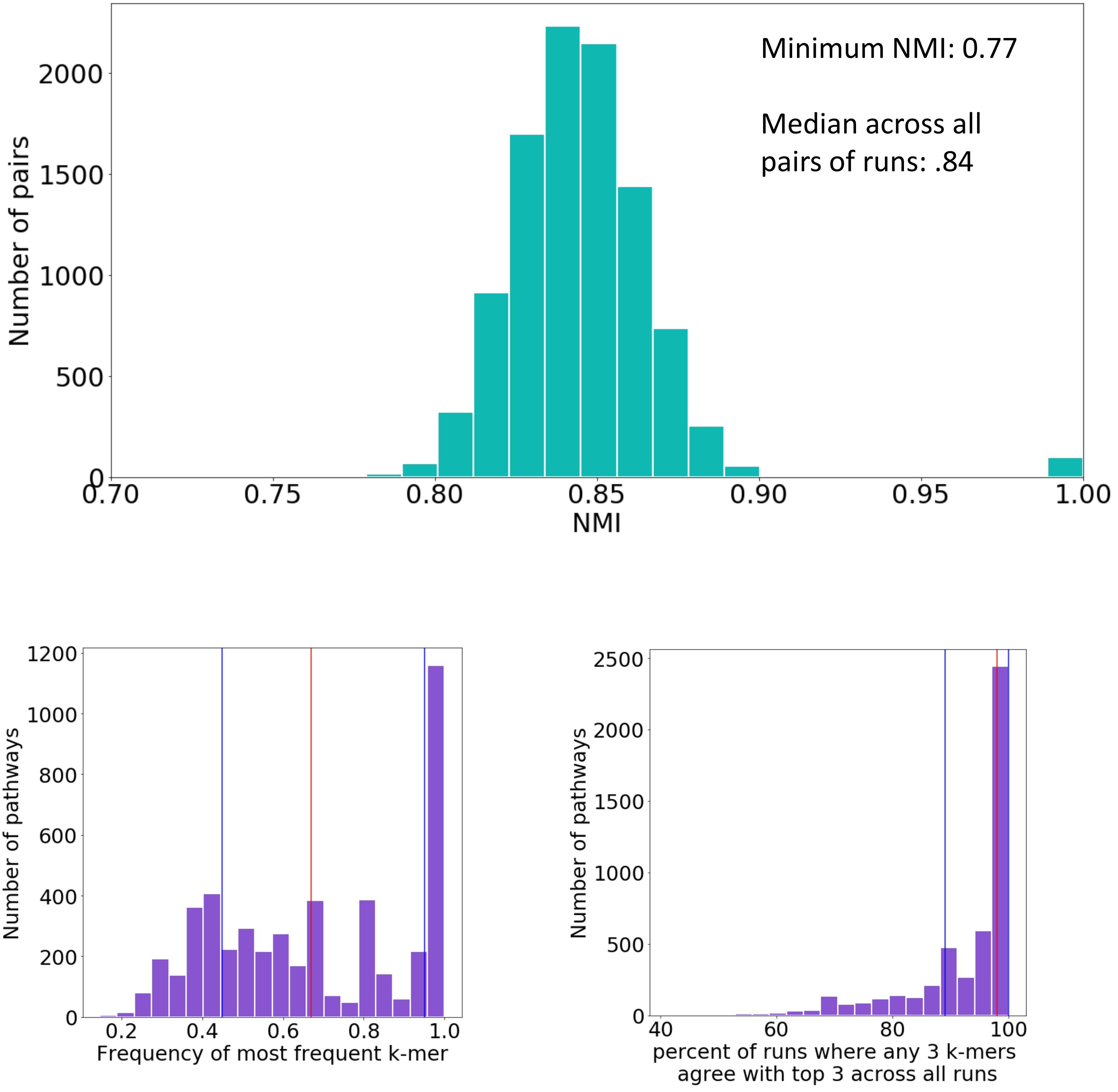

Supplementary Figure 6. Comparison of Louvain community detection results across random runs of different resolutions. We re-ran the Louvain algorithm ten times for resolutions ranging from 0.1 to 1. The heatmap indicates pairwise normalized mutual information between community assignments from these different runs. Red lines indicate resolutions for the associated block of random runs. Listed below the resolution, we also indicate the average number of communities (+/- standard deviation) for each resolution parameter evaluated.

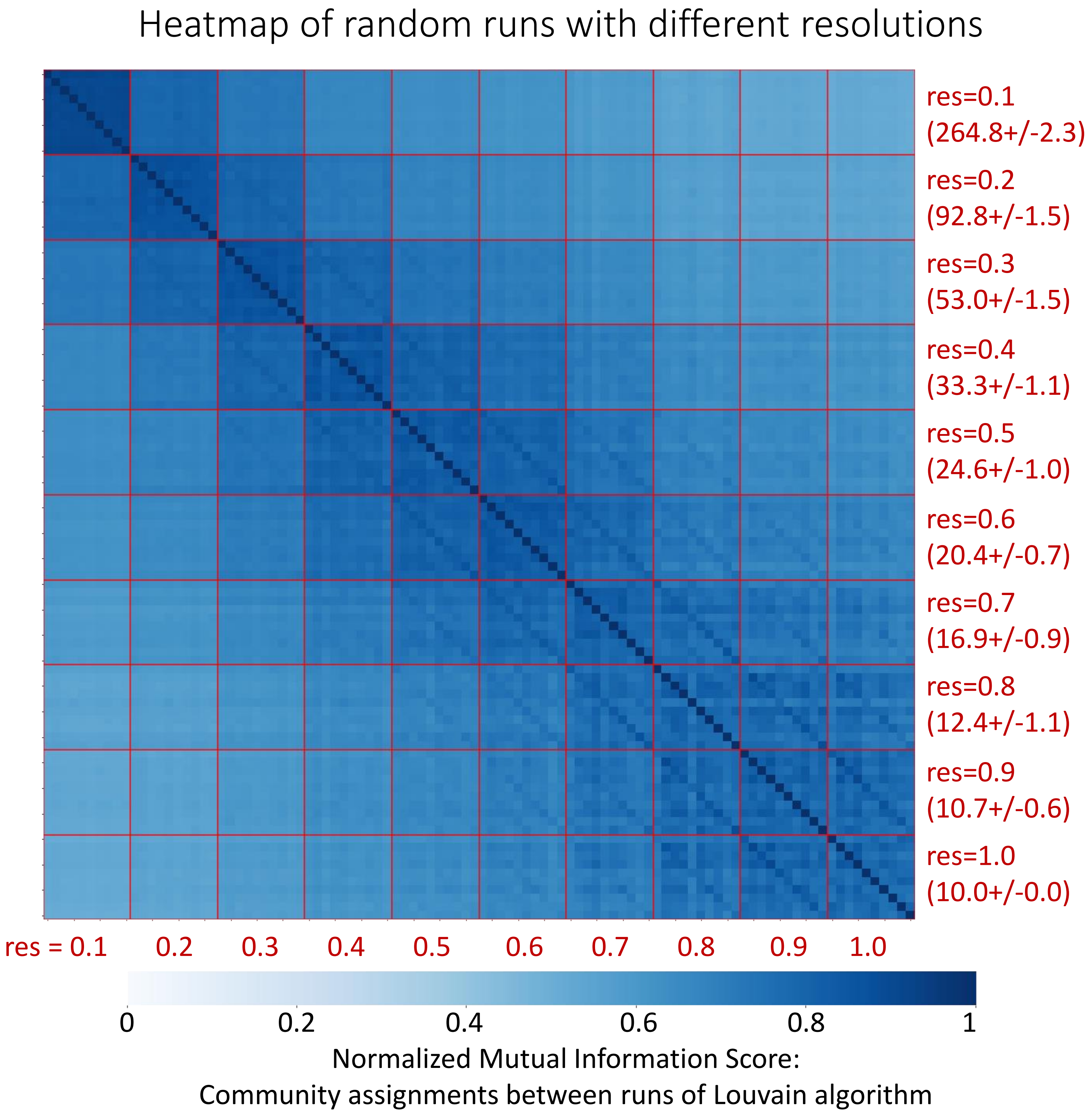

Supplementary Figure 7. Example of automatic label generation for Community 28. (A) Overview of pathways in Community 28 (as shown in Figure 4B). (B) Examples of how pathway names are converted to k-mer terms. (C) K-mer terms are gathered from each pathway, and within-community hubness is recorded for each pathway. (D) We aggregate unique k-mer terms, computing the number of times it appears across the pathway names, and average hubness of those pathways. (E) Our rank our final k-mers first by k, then count (filtering out those appearing in fewer than 3 pathways), and finally break ties with average hubness of the pathways containing the label.

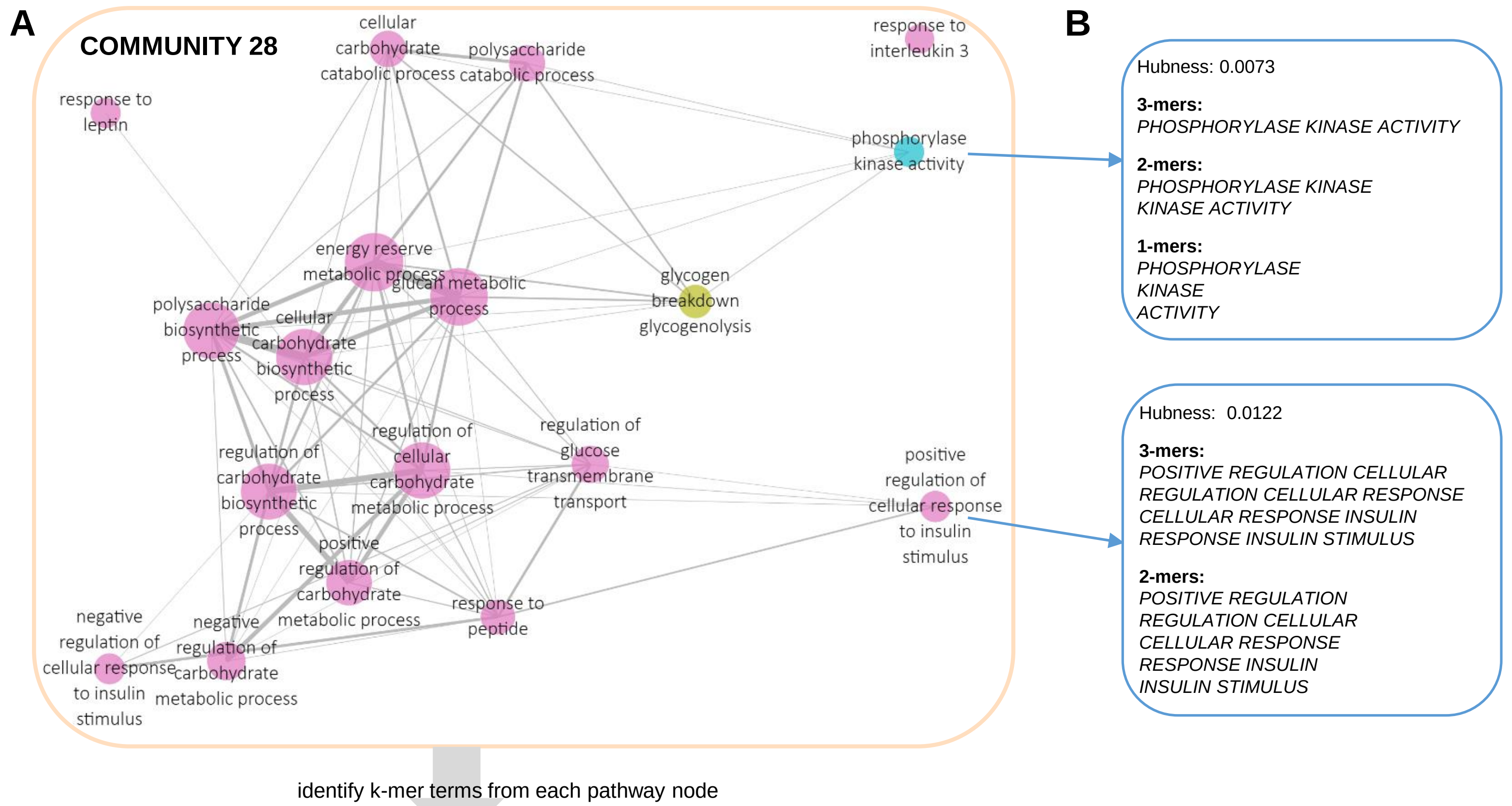

C

3-Mers (k=3)

pathway	term	hubness
GO_REGULATION_OF_CARBOHYDRATE_BIOSYNTHETIC_PROCESS	CARBOHYDRATE BIOSYNTHETIC PROCESS	0.11
GO_CELLULAR_CARBOHYDRATE_BIOSYNTHETIC_PROCESS	CARBOHYDRATE BIOSYNTHETIC PROCESS	0.11
GO_CELLULAR_CARBOHYDRATE_CATABOLIC_PROCESS	CARBOHYDRATE CATABOLIC PROCESS	0.03
GO_POSITIVE_REGULATION_OF_CARBOHYDRATE_METABOLIC_PROCESS	CARBOHYDRATE METABOLIC PROCESS	0.07
GO_NEGATIVE_REGULATION_OF_CARBOHYDRATE_METABOLIC_PROCESS	CARBOHYDRATE METABOLIC PROCESS	0.04
GO_REGULATION_OF_CELLULAR_CARBOHYDRATE_METABOLIC_PROCESS	CARBOHYDRATE METABOLIC PROCESS	0.11
GO_CELLULAR_CARBOHYDRATE_BIOSYNTHETIC_PROCESS	CELLULAR CARBOHYDRATE BIOSYNTHETIC	0.11
GO_CELLULAR_CARBOHYDRATE_CATABOLIC_PROCESS	CELLULAR CARBOHYDRATE CATABOLIC	0.03
GO_REGULATION_OF_CELLULAR_CARBOHYDRATE_METABOLIC_PROCESS	CELLULAR CARBOHYDRATE METABOLIC	0.11
GO_NEGATIVE_REGULATION_OF_CELLULAR_RESPONSE_TO_INSULIN_STIMULUS	CELLULAR RESPONSE INSULIN	0.01
GO_POSITIVE_REGULATION_OF_CELLULAR_RESPONSE_TO_INSULIN_STIMULUS	CELLULAR RESPONSE INSULIN	0.01

⋮

2-Mers (k=2)

pathway	term	hubness
GO_POLYSACCHARIDE_BIOSYNTHETIC_PROCESS	BIOSYNTHETIC PROCESS	0.11
GO_REGULATION_OF_CARBOHYDRATE_BIOSYNTHETIC_PROCESS	BIOSYNTHETIC PROCESS	0.11
GO_CELLULAR_CARBOHYDRATE_BIOSYNTHETIC_PROCESS	BIOSYNTHETIC PROCESS	0.11
REACTOME_GLYCOGEN_BREAKDOWN_GLYCOGENOLYSIS	BREAKDOWN GLYCOGENOLYSIS	0.02
GO_REGULATION_OF_CARBOHYDRATE_BIOSYNTHETIC_PROCESS	CARBOHYDRATE BIOSYNTHETIC	0.11
GO_CELLULAR_CARBOHYDRATE_BIOSYNTHETIC_PROCESS	CARBOHYDRATE BIOSYNTHETIC	0.11
GO_CELLULAR_CARBOHYDRATE_CATABOLIC_PROCESS	CARBOHYDRATE CATABOLIC	0.03
GO_POSITIVE_REGULATION_OF_CARBOHYDRATE_METABOLIC_PROCESS	CARBOHYDRATE METABOLIC	0.07
GO_NEGATIVE_REGULATION_OF_CARBOHYDRATE_METABOLIC_PROCESS	CARBOHYDRATE METABOLIC	0.04
GO_REGULATION_OF_CELLULAR_CARBOHYDRATE_METABOLIC_PROCESS	CARBOHYDRATE METABOLIC	0.11
GO_POLYSACCHARIDE_CATABOLIC_PROCESS	CATABOLIC PROCESS	0.03
GO_CELLULAR_CARBOHYDRATE_CATABOLIC_PROCESS	CATABOLIC PROCESS	0.03
GO_CELLULAR_CARBOHYDRATE_CATABOLIC_PROCESS	CELLULAR CARBOHYDRATE	0.03
GO_CELLULAR_CARBOHYDRATE_BIOSYNTHETIC_PROCESS	CELLULAR CARBOHYDRATE	0.11
GO_REGULATION_OF_CELLULAR_CARBOHYDRATE_METABOLIC_PROCESS	CELLULAR CARBOHYDRATE	0.11

⋮

D

term	Avg. hubness	count
CARBOHYDRATE BIOSYNTHETIC PROCESS	0.11	2
CARBOHYDRATE CATABOLIC PROCESS	0.03	1
CARBOHYDRATE METABOLIC PROCESS	0.07	3
CELLULAR CARBOHYDRATE BIOSYNTHETIC	0.11	1
CELLULAR CARBOHYDRATE CATABOLIC	0.03	1
CELLULAR CARBOHYDRATE METABOLIC	0.11	1
CELLULAR RESPONSE INSULIN	0.01	2

⋮

term	Avg. hubness	count
BIOSYNTHETIC PROCESS	0.11	3
BREAKDOWN GLYCOGENOLYSIS	0.03	1
CARBOHYDRATE BIOSYNTHETIC	0.07	2
CARBOHYDRATE CATABOLIC	0.11	1
CARBOHYDRATE METABOLIC	0.03	3
CATABOLIC PROCESS	0.11	2
CELLULAR CARBOHYDRATE	0.01	3

⋮

-Keep only labels with 3+ appearances
-Sort by: k, count, avg. hubness

E

label	k	count	Avg. hubness
CARBOHYDRATE METABOLIC PROCESS	3	3	0.075
METABOLIC PROCESS	2	5	0.093
BIOSYNTHETIC PROCESS	2	3	0.113
CELLULAR CARBOHYDRATE	2	3	0.087
CARBOHYDRATE METABOLIC	2	3	0.075
REGULATION CARBOHYDRATE	2	3	0.074
REGULATION CELLULAR	2	3	0.045
PROCESS	1	10	0.087
REGULATION	1	7	0.056
CARBOHYDRATE	1	6	0.08

Supplementary Figure 8. Pathway membership for the top 243 genes with highest differential expression between ER+ and ER- breast cancer samples in the METABRIC dataset. The genes are sorted from lowest to highest p-value (top to bottom, and then left to right). We additionally indicate whether the gene appears in any pathways (split by community). Gray squares on the heat map indicate communities in which the gene is significantly overrepresented, based on a one way chi square test (p<.05)

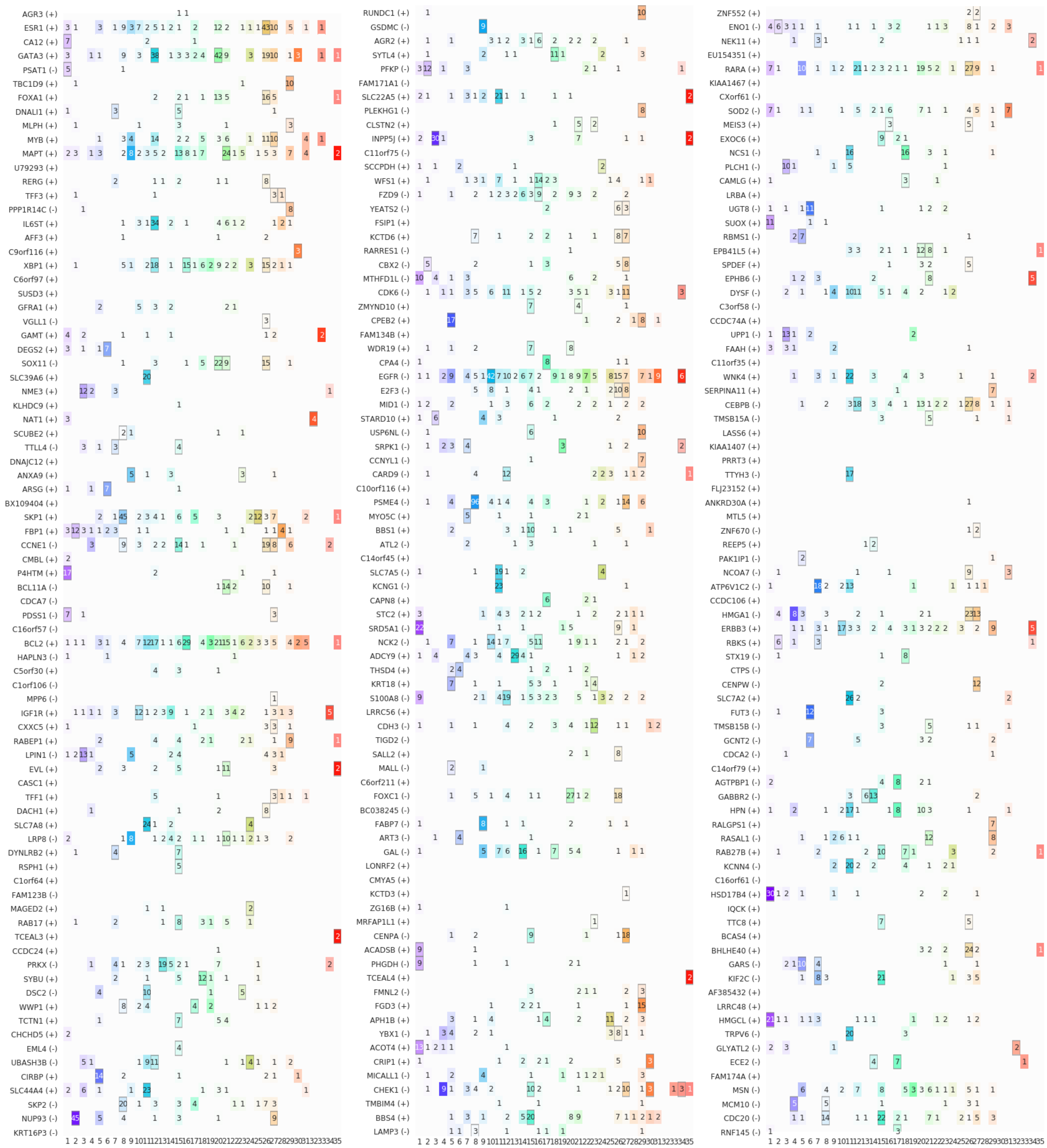

Supplementary Figure 9. Clustering consistency between final communities (learned across all 4 pathway databases) and curated KEGG categories. Cells are colored by the percent of curated category's pathway in each community; cell text indicates the exact number of pathways in each community and KEGG category (see Supplementary Figure 13 to see these results in the context of all databases).

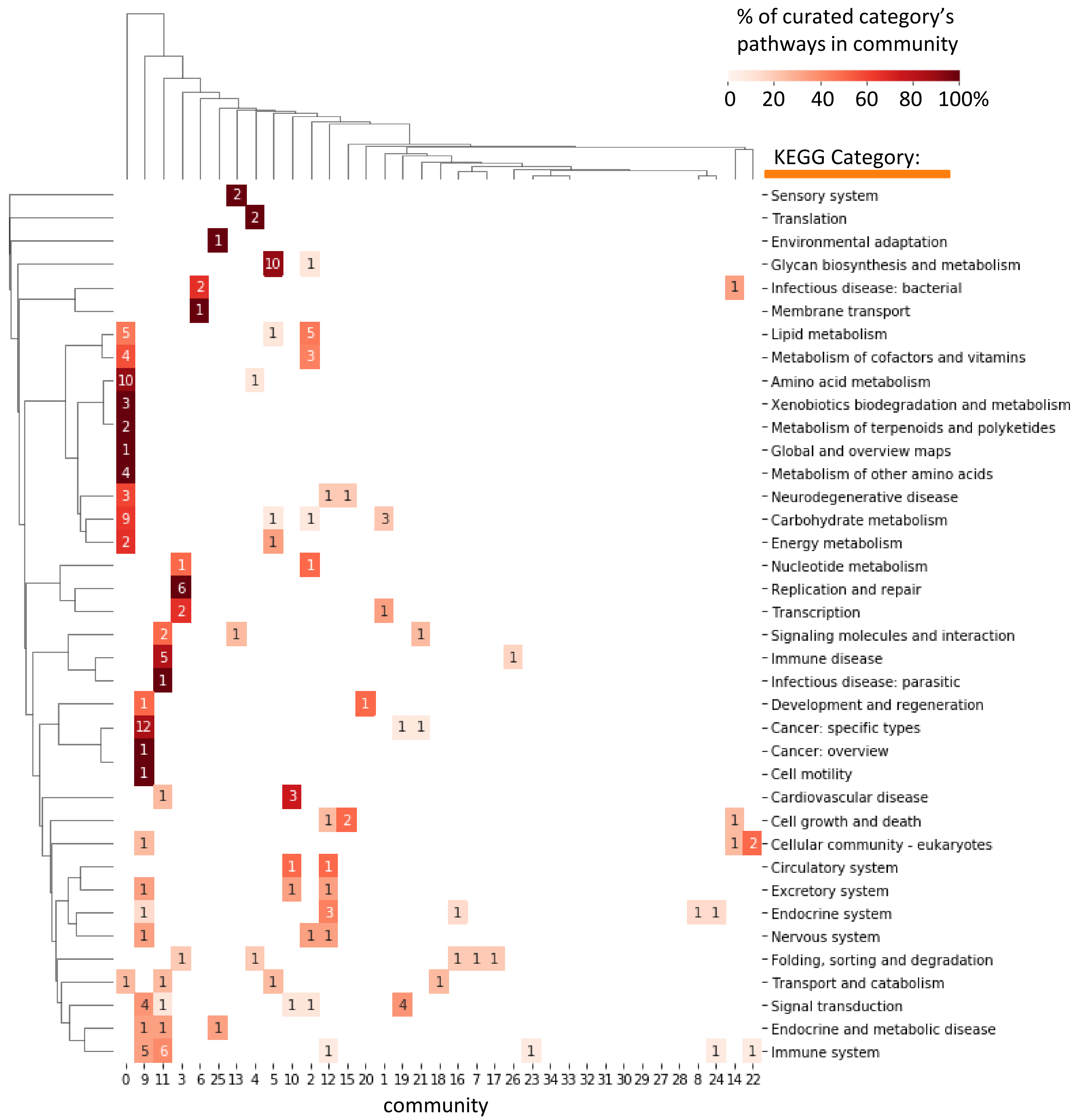

Supplementary Figure 10. Clustering consistency between final communities (learned across all 4 pathway databases) and curated REACTOME categories. Cells are colored by the percent of curated category's pathway in each community; cell text indicates the exact number of pathways in each community and REACTOME category (see Supplementary Figure 13 to see these results in the context of all databases).

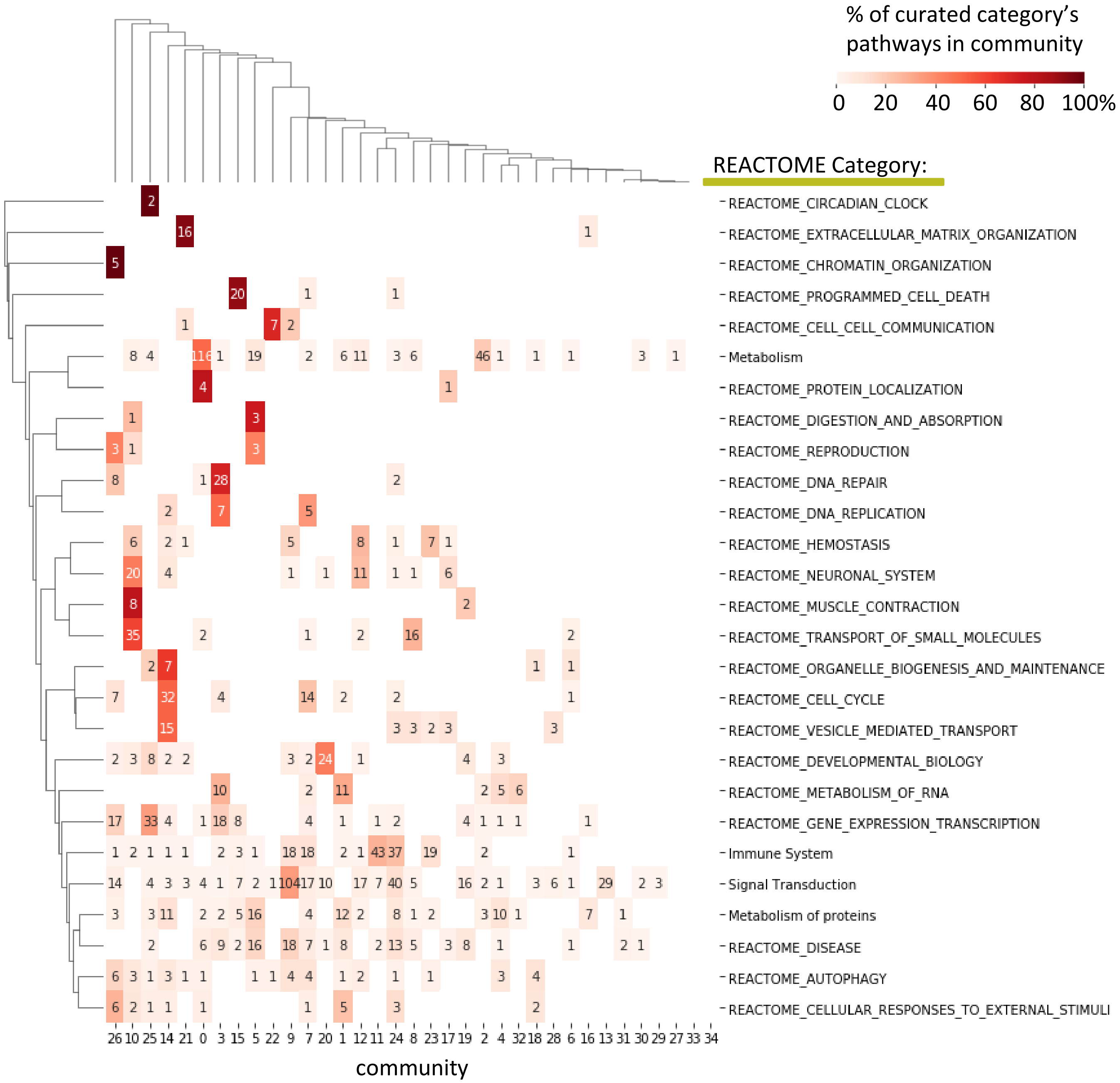

Supplementary Figure 11. Clustering consistency between final communities (learned across all 4 pathway databases) and curated GO BP categories. Cells are colored by the percent of curated category's pathway in each community; cell text indicates the exact number of pathways in each community and GO BP category (see Supplementary Figure 13 to see these results in the context of all databases).

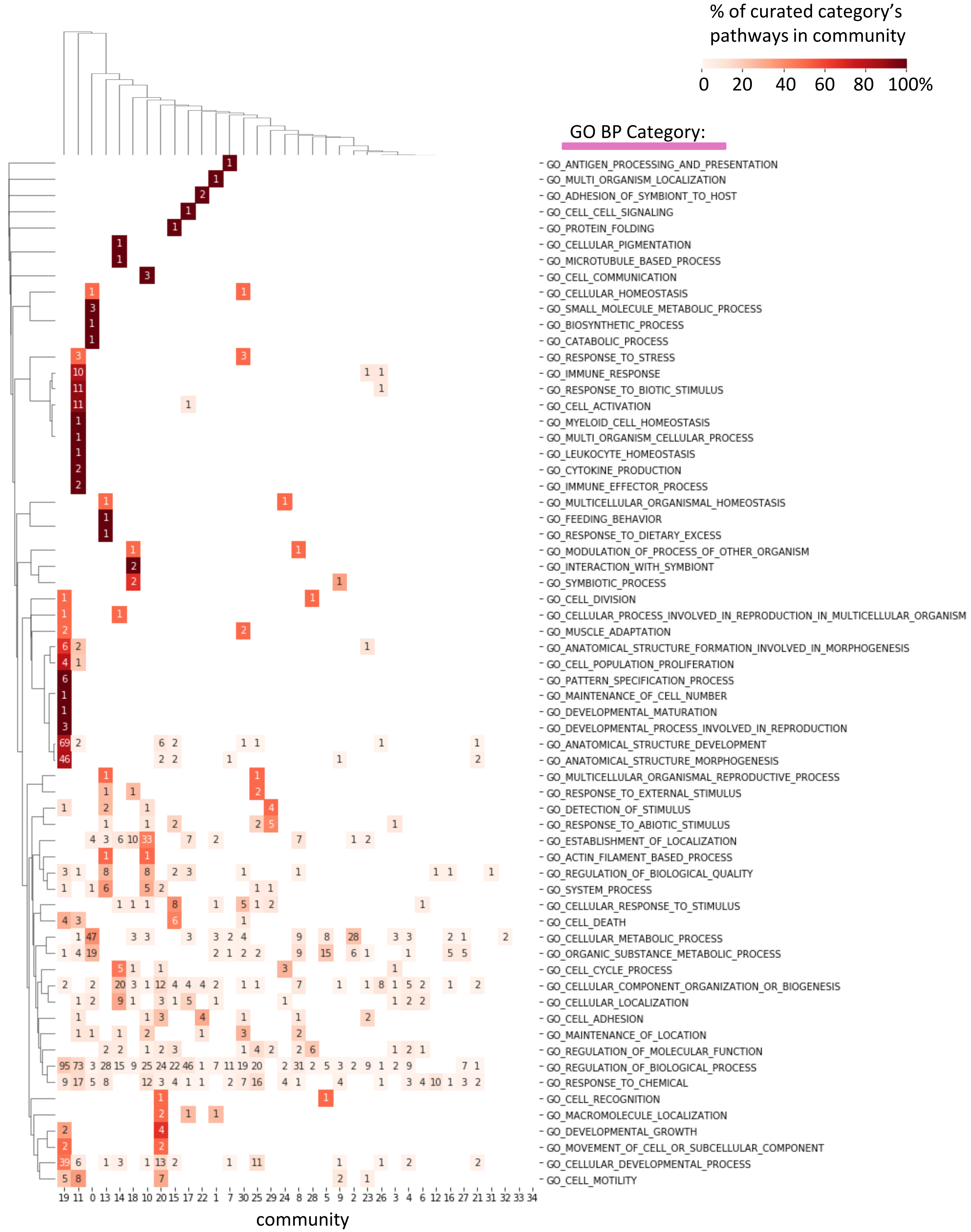

Supplementary Figure 12. Clustering consistency between final communities (learned across all 4 pathway databases) and curated GO MF categories. Cells are colored by the percent of curated category's pathway in each community; cell text indicates the exact number of pathways in each community and GO MF category (see Supplementary Figure 13 to see these results in the context of all databases).

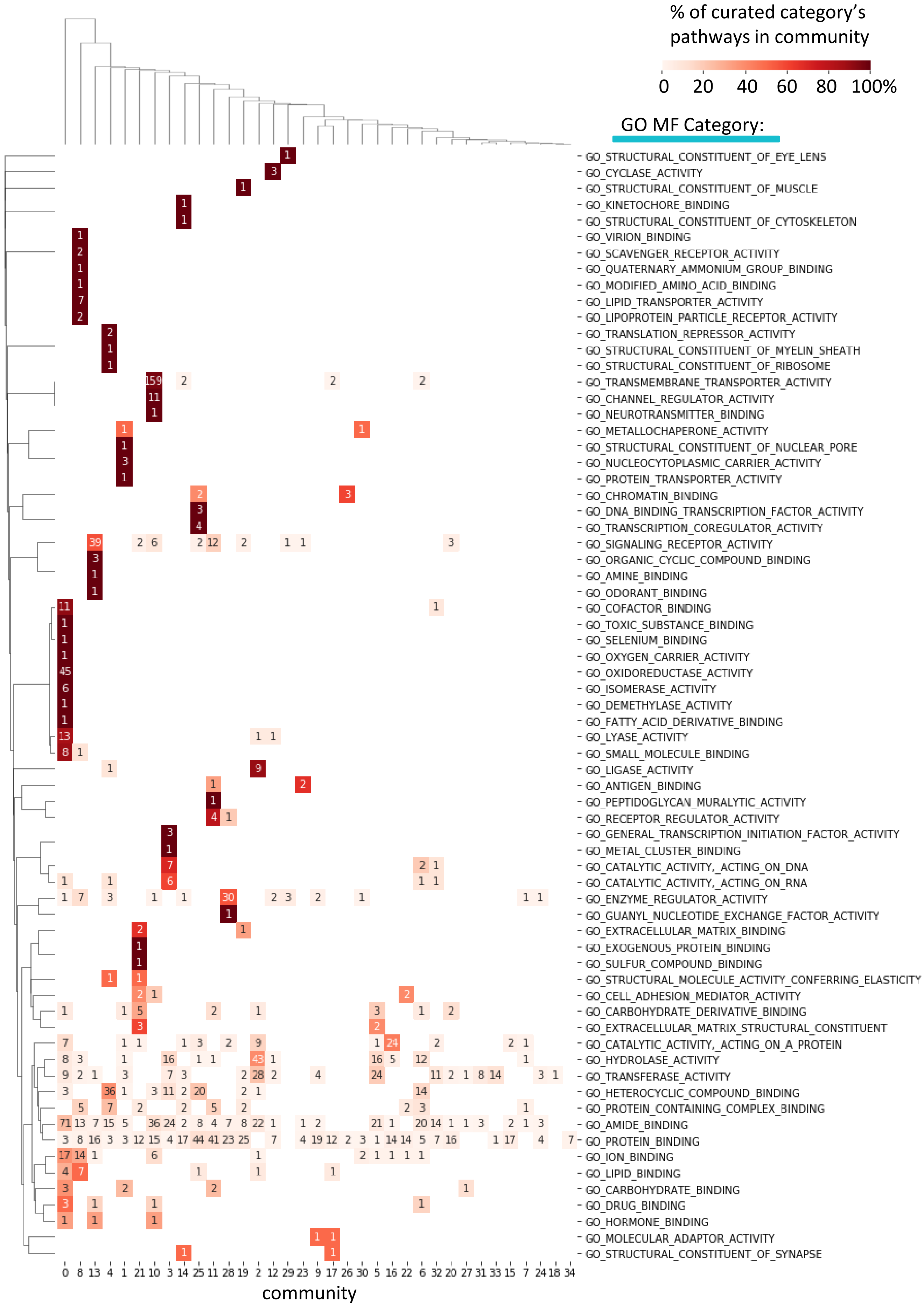

Supplementary Figure 13. Clustering consistency between final communities (columns) and all curated categories (rows) across KEGG, REACTOME, GO BP, and GO MF. Cells are colored by the percent of curated category's pathway in each community; cell text indicates the exact number of pathways in each community and category.

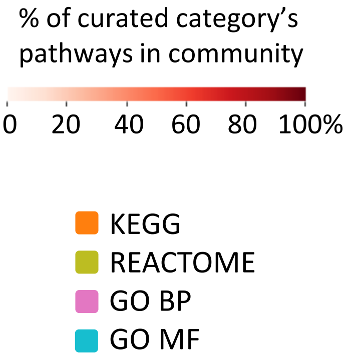

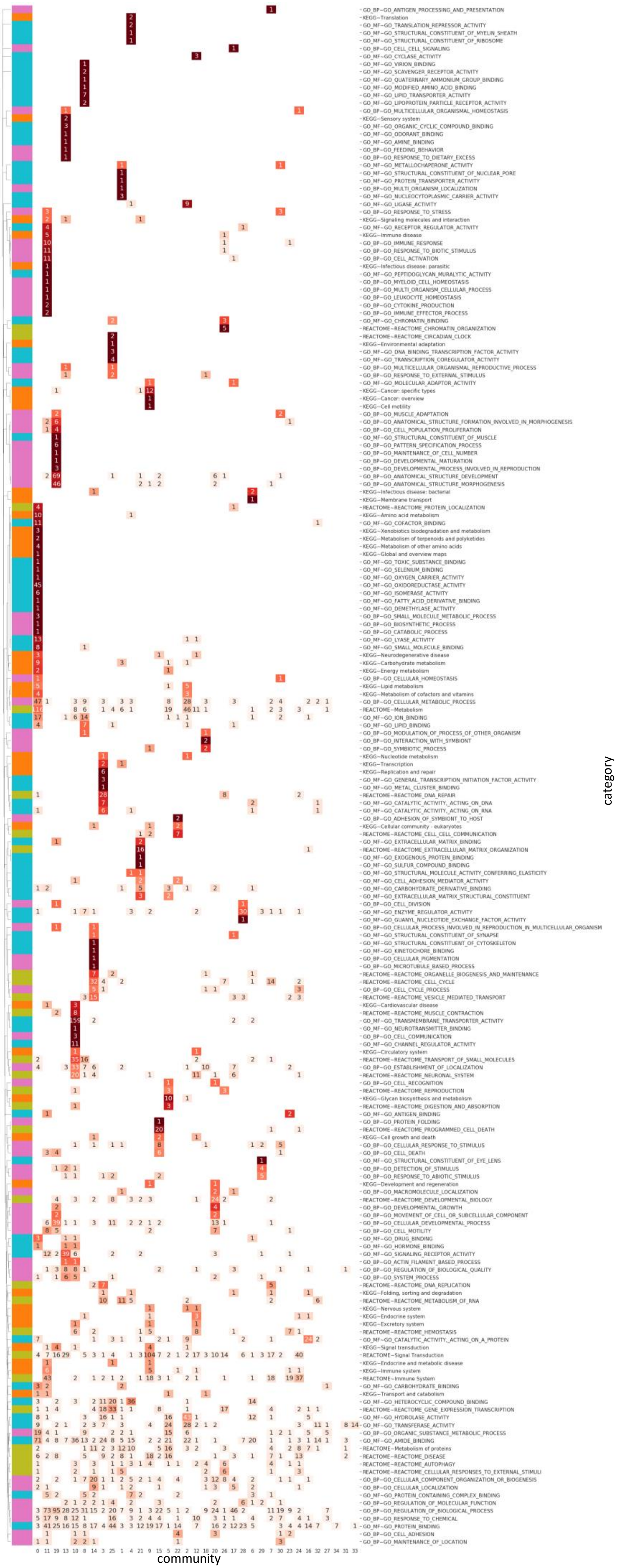

Supplement: lqac044_Supplemental_Files [file lqac044_supplemental_files.zip › SupplementaryMaterialsClean.pdf]
